# Trauma and social pathways to psychosis: Examining the role of attachment, social rank and dissociation in a clinical sample

**DOI:** 10.1111/bjc.12511

**Published:** 2024-10-29

**Authors:** Shelley Grady, Niall Crowley, Seamus Scott, Charles Ifegwu Ndukwe, Rebecca Donohoe, Keith Gaynor

**Affiliations:** ^1^ School of Psychology University College Dublin Belfield Ireland; ^2^ Adult Mental Health Services, Health Service Executive, CHO8 Longford/Westmeath Tullamore Ireland; ^3^ DETECT, Early Intervention in Psychosis Service Blackrock Ireland

**Keywords:** attachment, dissociation, mechanisms, pathways, psychosis, social rank, trauma

## Abstract

**Objectives:**

The trauma and social pathways model of psychosis proposes interactions between trauma, attachment, social rank and dissociation in pathways to psychosis, though this model has yet to be empirically investigated. The primary aim of this study was to examine the overall predictive value of the trauma and social pathways model using regression analysis. A secondary aim was to delineate hypothesized pathways between trauma and positive symptoms of psychosis using serial mediation analysis.

**Method:**

This was a cross‐sectional study of people attending mental health services for a psychosis‐related diagnosis (*N* = 71). Measures of trauma, positive symptoms of psychosis, attachment, social comparison and dissociation were completed.

**Results:**

A model of recurrent trauma, insecure attachment, social rank and dissociation predicted 23.2% of the variance in positive symptoms of psychosis. Recurrent trauma, attachment and dissociation contributed significantly to the model, while social rank did not. Further, serial mediation analysis indicated that the sequence of disorganized attachment and dissociation fully mediated the relationship between recurrent trauma and positive symptoms.

**Conclusions:**

Results provide preliminary support for the trauma and social pathways model of psychosis, specifically as it relates to recurrent trauma, insecure attachment and dissociation. Results did not support the social rank component of this model, however. These findings provide clear targets for the development of next‐wave psychological interventions that focus on trauma‐related variables in psychosis. Future studies should replicate these findings with a larger clinical sample, and consider a measure of shame to further elucidate social processes in pathways to psychosis.

**Practitioner points**

- Insecure attachment and dissociation may be important mechanisms by which recurrent trauma leads to symptoms of psychosis in people with psychosis‐related diagnoses.
- Therapeutic interventions for people with psychosis should attend to attachment and dissociative processes, and compassion‐focused therapy may be particularly well suited to do this.
- Attachment security can be fostered at a service level by attuning to people's pattern of engagement with mental health teams, and promoting safeness through warmth, sensitivity and consistency in care.

## INTRODUCTION

The causal relationship between trauma and psychosis is now widely recognized (Bailey et al., 2018; Bentall et al., 2012; de Vries et al., 2019; Read et al., 2009; Varese, Barkus, & Bentall, 2012; Varese, Smeets, et al., 2012). Our understanding of the pathways that lead from trauma to psychosis remains limited, however, and relies mainly on the measurement of psychotic‐like experiences in general population studies (Bloomfield et al., 2021; Williams et al., 2018). To determine the factors to be targeted in therapeutic interventions, we need to improve our understanding of these pathways, particularly among people with clinically significant experiences of psychosis.

Dissociation is one of the best‐evidenced mediators of the trauma–psychosis relationship to date (Bloomfield et al., 2021; Williams et al., 2018). It is an umbrella term that refers to a range of detachment (depersonalization, derealization) and compartmentalization (suppression of thoughts and emotions) strategies. Dissociation is widely recognized as an adaptive response to trauma to avoid the integration of negative experiences and mitigate distress (Liotti & Gumley, 2008). Meta‐analyses have demonstrated robust positive associations between dissociation and positive symptoms of psychosis (Longden et al., 2020; Pilton et al., 2015), though the theoretical underpinnings of this association are debated. Some researchers argue that most, if not all, positive symptoms can be explained by a combination of dissociated identity, affect and behaviour, depersonalization, derealization and flashbacks (Moskowitz et al., 2009), while others have instead focused on specific mechanisms that may explain the relationship (e.g., cognitive biases; Varese, Barkus, & Bentall, 2012; Varese, Smeets, et al., 2012).

Given its relevance to interpersonal trauma, attachment has been a topic of significant investigation in psychosis (Berry et al., 2008; Liotti & Gumley, 2008). According to attachment theory (Bowlby., 1973), early caregiving relationships lead to the development of internal working models of the self and others in relationships, which manifest in patterns of attachment. When caregivers are attuned to a child's needs, they are more likely to develop a secure attachment pattern (Ainsworth et al., 1978). When caregivers are not able to meet attachment needs, an insecure pattern is likely to develop. An insecure anxious attachment pattern is characterized by an increase in attachment behaviours in an attempt to ensure care from an inconsistent caregiver, while an avoidant pattern involves a decrease in attachment behaviours in an attempt to maintain closeness to a rejecting caregiver. A disorganized pattern is characterized by oscillations between anxious and avoidant patterns, and is thought to arise in response to frightening or frightened caregivers (Main and Solomon, 1986). The child is unable to organize their behaviour because their primary attachment figure is both a source of safety and distress (Liotti, 2004).

Insecure attachment patterns are overrepresented in psychosis populations (76% compared to 38% in non‐clinical samples) and there is meta‐analytic evidence of small but significant positive associations between insecure attachment and positive symptom severity (Carr et al., 2018a). When it comes to elucidating specific patterns of insecure attachment in psychosis, the literature is somewhat mixed. While Gumley et al. (2014) concluded good evidence to support the relationship between avoidant attachment and positive symptoms, and modest evidence for anxious attachment, a more recent review (Carr et al., 2018a) concluded stronger evidence for anxious attachment than avoidant. Similarly, Lavin et al. (2020) examined the relationship between insecure attachment and paranoia specifically, and found that anxious attachment was more strongly associated with paranoia than avoidant. There has been less empirical research on disorganized attachment in psychosis (in part due to the lack of a validated measure until recently; Pollard et al., 2020), though emerging evidence suggests it may be a particularly robust mediator of the trauma–psychosis relationship (Grady et al., 2024). This is supported by attachment theorists, who propose that one of the ways that disorganized attachment may exert its influence on psychosis is by amplifying dissociative processes, in which the individual is unable to integrate thoughts, emotions, and experiences into consciousness, memory and self‐identity (Berry et al., 2017; Liotti, 2004; Liotti & Gumley, 2008).

As the definition of trauma has expanded to include psychologically traumatizing events (ICD‐11), there has been increased recognition of the impact of broader social experiences. Robust research now points to the role of urbanicity (Vassos et al., 2012), isolation (Michalska Da Rocha et al., 2018), perceived discrimination (Pearce et al., 2019) and migration (Cantor‐Graae & Selten, 2005) in the development of psychosis. These experiences all share commonalities of low social rank, which has led researchers to question the role that social rank might play in pathways between trauma and psychosis (Heriot‐Maitland et al., 2022). Social rank theory (Gilbert & Allan, 1998) describes how humans have evolved to coordinate social living, whereby low‐rank members are subordinate to high‐rank members and group cohesion is achieved by social mentalities (patterns of mental organization, behaviour). For example, low‐rank mentalities might include perceiving oneself as inferior and behaving submissively.

Although under‐researched, there is some experimental evidence for the role of social rank in psychosis. A series of studies have demonstrated that voice‐hearing reflects perceptions of low social rank (Birchwood et al., 2004; Gilbert et al., 2001) and it has been associated with paranoia in non‐clinical populations (Freeman et al., 2005). Further, low social rank is commonly associated with feelings of shame, and meta‐analytic evidence indicates a moderate to strong relationship between shame and positive symptoms of psychosis (Carden et al., 2020). One recognized way that people try to regulate feelings of shame is dissociation (Dorahy & Clearwater, 2012; Talbot et al., 2004), and several studies suggest that shame may play a role in the development of both dissociative and psychotic‐like experiences (Dorahy et al., 2017; Dorahy & Clearwater, 2012; Kouri et al., 2023; Matos et al., 2013).

In a recently proposed model, Heriot‐Maitland et al. (2022) build on existing theories of trauma and psychosis that point to the role of attachment and dissociative processes (Berry et al., 2017; Hardy, 2017) by highlighting the potential relevance of wider social processes (i.e., social rank) in these pathways. They point to interpersonal trauma as a determinant for both attachment and social rank patterns, and dissociation as a potential consequence of both, suggesting that some, or all, of these mechanisms may interact in pathways to psychosis.

The primary aim of this study was to test the overall predictive value of a model of trauma, attachment, social rank and dissociation on positive symptoms of psychosis in people with a psychosis‐related diagnosis. A secondary aim was to delineate specific pathways by which trauma may lead to positive symptoms. In line with theoretical accounts and emerging evidence that highlights the role of disorganized attachment (Berry et al., 2017; Grady et al., 2024; Liotti, 2004), we hypothesized that the sequence of disorganized attachment and dissociation would mediate the relationship between trauma and (i) total positive symptoms (ii) hallucinations (iii) delusions. Further, we hypothesized that the sequence of social rank and dissociation would mediate the relationship between trauma and (i) total positive symptoms (ii) hallucinations (iii) delusions.

## METHOD

### Research design and participants

This study was pre‐registered on Open Science Framework (https://osf.io/f5bz8/). The pre‐registration protocol covered both the current study and a complementary study focused on negative symptoms in psychosis.

This was a cross‐sectional study in which participants were recruited across four geographically‐defined community mental health services in the HSE (Ireland's public health service). This included two secondary care and two tertiary care services for people experiencing longer‐term difficulties with community living. Inclusion criteria included clients aged 18–65 years with a psychosis‐related diagnosis, capacity to provide informed consent, and sufficient command of English to engage in the assessment. DSM‐V diagnoses were confirmed by treating teams and clients were excluded if their psychosis was deemed organic in nature. Eligible clients were identified by clinicians and approached about the study by a research gatekeeper.

### Procedure and measures

Individuals willing to take part met with the researcher to discuss the study, obtain informed consent and complete the below measures. Researchers completing data collection included an advanced nurse practitioner (S.S.), a non‐consultant hospital doctor (C.I.N.) and a doctoral student in clinical psychology (S.G.). SAPS training was provided by S.S. and inter‐rater reliability was achieved between researchers prior to data collection using a consensus training model. For first‐episode psychosis (FEP) participants, the SAPS was completed by S.S. as part of routine clinical care and these ratings were used for research purposes with the participant's consent.

### Measures

#### Scale for the assessment of positive symptoms (SAPS; Andreasen, 1984)

The SAPS is an interview‐based measure of four major positive symptoms: hallucinations, delusions, bizarre behaviour and positive formal thought disorder. Higher scores on each item indicate greater symptom severity, ranging from 0 = ‘Not at all’ to 5 = ‘Severe’. The current study used the four global symptom domain items only, with total scores ranging from 0 to 20. The SAPS has been shown to have good criterion and factorial validity, but questionable internal consistency (*α* = .40; Andreasen, 1984; Peralta & Cuesta, 1999), likely due to the heterogeneous nature of positive symptoms. It demonstrated acceptable levels of internal consistency in the current study (*α* = .70).

#### Dissociative experiences scale‐revised (DES‐II; Carlson & Putnam, 1993)

The DES‐II measures dissociative experiences across three subscales: depersonalization and derealization; amnesia; and absorption. Respondents indicate the percentage of time they experience each item on a scale of 0%–100%. Total scores are determined by calculating the average score for all items. The DES‐II has demonstrated high internal consistency (*α* = .95; Carlson & Putnam, 1993) and is widely used to measure dissociation in people with psychosis (e.g., Degnan et al., 2022). In the current study, item 27, which pertains to hearing voices, was removed for formal analysis. The 27‐item DES‐II demonstrated high levels of internal consistency (*α* = .93).

#### Trauma and life events checklist (TALE; Carr et al., 2018b)

The TALE measures exposure to adverse life experiences that are commonly reported by people with psychosis. It has shown good psychometric acceptability overall (Carr et al., 2018b), with excellent convergent validity and reliability for sexual abuse. The current study used items 1–19 of Part A, in which respondents are asked about exposure to specific life events. For each item endorsed, participants are asked if the event occurred more than once and at what age. A total trauma score was calculated by adding all events endorsed, and a recurrent trauma score was calculated by adding events that occurred more than once. Reliability analysis yielded acceptable levels of internal consistency (total trauma *α* = .78; recurrent trauma *α* = .77).

#### Psychosis attachment measure‐revised (PAM‐R; Pollard et al., 2020)

The PAM‐R is a measure of attachment patterns in psychosis, adapted from the original PAM (Berry et al., 2006) to include a disorganized attachment subscale. Participants rate the degree to which each statement is like them on a scale from 0 to 3, and three subscales are obtained: anxious, avoidant and disorganized. The PAM‐R has previously demonstrated high levels of internal consistency (anxious *α* = .87, avoidant *α* = .79, disorganized *α* = .89). In the current study, subscales demonstrated questionable to high internal consistency (anxious *α* = .78, avoidant *α* = .62, disorganized *α* = .88).

#### Social comparison scale (SCS; Allan & Gilbert, 1995)

The SCS is a widely used measure of social rank (e.g., Cheung et al., 2004) that has previously been used with people with psychosis (Wood & Irons, 2016). Respondents rate themselves from 0 to 10 in relation to others using a series of bipolar constructs (e.g., insider–outsider), with total scores ranging from 0 to 110. Lower scores reflect lower levels of social rank. It has good psychometric properties (Allan & Gilbert, 1995) and has previously demonstrated high internal consistency (*α* = .91). It demonstrated similarly high levels in the current study (*α* = .93).

### Data analysis

Data were analysed using SPSS v.27. There was less than 2% missing data (Missing Completely at Random), which was dealt with by expectation maximization (Do & Batzoglou, 2008). Two variables (DES‐II and SAPS hallucinations) were positively skewed and transformed using square root transformations. Following this, all data met assumptions for normality, linearity, multi‐collinearity and homoscedasticity. Preliminary analyses (*t*‐tests, ANOVAS, Pearson's correlations) were completed to (i) identify differences in outcomes by sociodemographic and clinical characteristics and (ii) identify bivariate associations between measures. To test the primary hypothesis, hierarchical linear regression was conducted to examine the overall predictive value of the proposed model. To test the secondary hypotheses, serial mediation analyses were conducted to delineate proposed pathways using the PROCESS macro v.4.2. (Hayes, 2022). The statistical significance of the indirect effects was tested using bootstrapped bias‐corrected percentile‐based confidence intervals of 5000 bootstrap draws.

### Power analysis

Based on prior research (Humphrey et al., 2022; Pearce et al., 2017), small to medium effect sizes were estimated between variables of interest. G*Power (Faul et al., 2007) indicated that 29 participants would be needed to detect a medium effect size (*d* = .50) with 95% power in a regression model with four predictor variables, while 68 participants would be needed to detect a small effect size (*d* = .20). Guidelines for mediation analyses (Sim et al., 2022) indicated that 50 participants would be needed to detect a medium effect size in a full or partial mediation using bootstrapping, while 110–130 participants would be needed to detect a small effect size.

## RESULTS

### Sample and descriptive characteristics

Table 1 presents the descriptive characteristics of the sample. The mean age of participants was 43 years (*SD* = 11.88); the majority were male (59.2%), white‐Irish (87.3%), single (77.5%) and not currently working (57%). The most frequently reported diagnosis was schizophrenia (39.4%) and the mean length of contact with mental health services for a psychosis‐related diagnosis was 14.6 years (*SD* = 12.13). Participants reported exposure to a mean of 8.15 (*SD* = 3.87) adverse life events on the TALE. Detailed rates of trauma and abuse types are provided in Tables S1–S13.

**TABLE 1:** Sociodemographic and clinical information (*N* = 71).

		Descriptive statistic
Age Mean (SD)		43 (11.88)
Range (years)		20–65
Gender (%)	Female	29 (40.8)
	Male	42 (59.2)
Ethnicity (%)	White‐Irish	62 (87.3)
	Non‐Irish and/or minority status	9 (12.7)
	*White‐Irish Traveller* *White‐Polish*	*3* *2*
	*Black*	*1*
	*Asian*	*1*
	*White‐English*	*1*
	*White‐American*	*1*
Relationship status (%)	Single	55 (77.5)
	In a relationship	16 (22.5)
Education level (%)	Junior certificate or equivalent or below^a^	16 (22.5)
	Leaving certificate or equivalent^b^	14 (19.7)
	Some college	26 (36.6)
	Undergraduate	8 (11.3)
	Post‐graduate or above	7 (9.9)
Employment (%)	Working/studying	30 (42.3)
	Unemployed	41 (57.7)
Diagnosis (%)	Schizophrenia	28 (39.4)
	Schizoaffective	14 (19.7)
	Delusional disorder	2 (2.8)
	First‐episode psychosis	15 (21.1)
	Psychosis (not otherwise specified)	3 (4.2)
	Depression with psychosis	4 (5.6)
	Bipolar with psychosis	5 (7)
Mental health service (%)	Community‐based secondary care	61 (85.9)
	Community‐based tertiary care	10 (14.1)
Length of contact with mental health services for psychosis‐related diagnosis Mean (SD)		176 months (145.56) 14.6 years (12.13)
Range		2 months to 41 years
Current medication (%)	Yes	67 (94.4)
	No	4 (5.6)
TALE Mean (SD)	Total items endorsed	8.15 (3.87)
	Recurrent trauma	5.86 (3.61)
SAPS Mean (SD)	Global total	4.07 (3.36)
	Global hallucinations	1.13 (1.42)
	Global delusions	1.42 (1.43)
PAM‐R Mean (SD)	Anxious	9.17 (4.68)
	Avoidant	9.05 (3.35)
	Disorganized	8.61 (6.02)
DES‐II Mean (SD)		17.68 (14.27)
SCS Mean (SD)		53.63 (19.89)

Abbreviations: DES‐II, Dissociative Experiences Scale‐II; PAM‐R, Psychosis Attachment Measure‐Revised; SAPS, Scale for the Assessment of Positive Symptoms; SCS, Social Comparison Scale; TALE, Traumatic and Adverse Life Events scale.

^a^ Irish state exams completed in the third year of second‐level education.

^b^ Irish state exams completed in the final year of second‐level education.

### Preliminary analyses

Preliminary analyses indicated no significant differences on primary measures based on relationship status, employment status or mental health service. Males (*M* = 1.74; *SD* = 1.45) scored significantly higher than females (*M* = 0.97; *SD* = 1.30) on measures of delusions, *t*(69) = −2.304, *p* = .024. While there were significant differences between measures based on ethnicity, age, level of education, medication, diagnoses and duration of contact with mental health services (see Tables S1–S13), these were due to exceptionally small n's within specific cells or small effect sizes and were not controlled for given the limited sample size.

Pearson's correlations (Table 2) indicated that social rank was negatively associated with anxious attachment, disorganized attachment and dissociation, but not avoidant attachment, trauma or psychosis. Anxious attachment was positively associated with trauma and dissociation, but not psychosis. Avoidant attachment was positively associated with psychosis, but not trauma or dissociation. The strongest correlations between proposed variables were between recurrent trauma, disorganized attachment, dissociation and psychosis.

**TABLE 2:** Correlation matrix.

	1	2	3	4	5	6	7	8	9	10
TALE total			0.209 (.080)	0.147 (.223)	0.108 (.372)	0.358** (.002)	−0.179 (.136)	0.218 (.068)	0.038 (.754)	0.289* (.015)
2 TALE recurrent			0.250* (.035)	0.119 (.321)	0.164 (.170)	0.318** (.007)	−0.182 (.128)	0.279* (.018)	0.042 (.723)	0.382** (.001)
3 SAPS total						0.475** (<.001)	−0.149 (.215)	0.139 (.247)	0.314** (.008)	0.300* (.011)
4 SAPS hallucinations						0.486** (<.001)	−0.153 (.203)	0.122 (.310)	0.300* (.011)	0.266* (.025)
5 SAPS delusions						0.435** (<.001)	−0.217 (.070)	0.154 (.200)	0.209 (.080)	0.258* (.030)
6 DES‐II							−0.289* (.014)	0.419** (<.001)	0.226* (.058)	0.480** (<.001)
7 SCS								−0.439** (<.001)	−0.162 (.176)	−0.464** (<.001)
8 PAM Anxious									0.155 (.197)	0.652*** (<.001)
9 PAM Avoidant										0.314** (.008)
10 PAM Disorganised										

*Note*: Figures in parentheses are exact *p*‐values.

* *p* < .05.

** *p* < .01.

*** *p* < .001.

### Hierarchical regressions

A four‐step hierarchical linear regression (Table 3) was conducted to examine the overall predictive value of a model of trauma and social pathways to psychosis, with SAPS total score as the dependent variable. SAPS total scores were significantly predicted by the model, adjusted *R* ^2^ = .232, *F*(1, 64) = 4.524 *p* < .001. Recurrent trauma, avoidant attachment and dissociation contributed significantly to the model, while anxious attachment, disorganized attachment and social rank did not. The model also predicted hallucinations (adjusted *R* ^2^ = .224, *F*(1, 64) = 14.558 *p* < .001.) and delusions (adjusted *R* ^2^ = .189, *F*(1, 63) = 4.524 *p* = .003) when each symptom domain was entered as the dependent variable (see Tables S1–S13).

**TABLE 3:** Hierarchical linear regression predicting SAPS total score.

Predictors	Adjusted *R* ^2^	Δ*R* ^2^	*B*	*SE B*	*b*	Model F (*df*)	*p*
**Step 1**	.049	.062*				4.60 (1, 69)	.035*
Recurrent trauma			.233	.109	.250*		
**Step 2**	.126	.113*				3.516 (4, 66)	.012**
Recurrent trauma			.172	.113	.185		
PAM anxious			−.061	.106	−.086		
PAM avoidant			.255	.119	.255*		
PAM disorganized			.115	.090	.205		
**Step 3**	.113	.000				2.777 (5, 65)	.025*
Recurrent trauma			.172	.114	.185		
PAM anxious			−.065	.109	−.091		
PAM avoidant			.255	.120	.254*		
PAM disorganized			.111	.093	.199		
Social rank			−.004	.022	−.022		
**Step 4**	.232	.122				4.524 (6, 64)	<.001***
Recurrent trauma			.110	.108	.118		
PAM anxious			−.117	.103	−.163		
PAM avoidant			.211	.112	.210		
PAM disorganized			.054	.088	.097		
Social rank			.020	−.001	−.007		
Dissociation			.510	.153	.412***		

*p < .05.

**p < .01.

***p < .001.

### Serial mediation

Serial mediation analyses were conducted to explore theorized relationships between variables of interest. In line with *a‐priori* hypotheses, three serial mediation models were estimated to (a) examine the indirect effect of recurrent trauma on total positive symptoms via a sequence of disorganized attachment and dissociation (b) examine the indirect effect of recurrent trauma on hallucinations via a sequence of disorganized attachment and dissociation (c) examine the indirect effect of recurrent trauma on delusions via a sequence of disorganized attachment and dissociation. Gender was controlled for in the delusion model, given that preliminary analyses indicated significant differences between groups. The second set of hypotheses that related to social rank as mediator were not examined, given that social rank was not associated with trauma or psychosis.

#### Total positive symptoms

**FIGURE 1:**
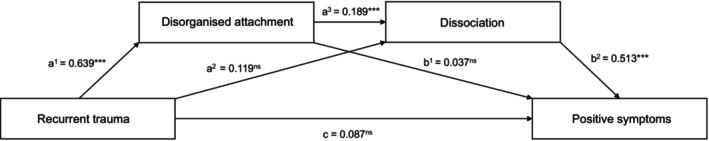
Mediation model testing if disorganized attachment and dissociation serially mediate the relationship between recurrent trauma and positive symptoms. ***p < .001; ^ns^ nonsignificant.

As shown in Figure 1, in the path recurrent trauma‐ > disorganized attachment‐ > positive symptoms, recurrent trauma had a significant positive effect on disorganized attachment (a^1^: *b* = 0.639, *p* = .001), while disorganized attachment did not have a significant effect on positive symptoms of psychosis (b^1^: *b* = 0.037, *p* = .605).

In the path recurrent trauma‐ > dissociation‐ > positive symptoms, recurrent trauma did not significantly affect dissociation (a^2^: *b* = 0.119, *p* = .169); however, dissociation had a significant positive effect on positive symptoms (b^2^: *b* = 0.513, *p* = .001).

In the path recurrent trauma‐ > disorganized attachment‐ > dissociation‐ > positive symptoms, recurrent trauma positively impacted disorganized attachment (a^1^: *b* = 0.639, *p* = .001), disorganized attachment positively impacted dissociation (a^3^: *b* = 0.189, *p* < .001) and dissociation positively impacted positive symptoms (b^2^: *b* = 0.513, *p* = .001). Furthermore, the direct effect of recurrent trauma on positive symptoms was not significant (c: *b* = 0.087, *p* = .429). This indicates that the sequence of disorganized attachment and dissociation fully mediated the relationship between recurrent trauma and positive symptoms of psychosis.

#### Hallucinations

**FIGURE 2:**
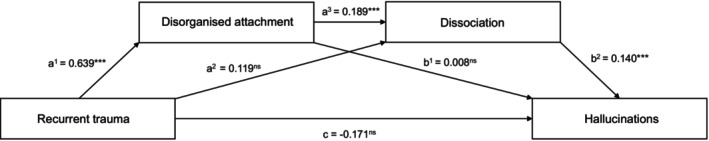
Mediation model testing if disorganized attachment and dissociation serially mediate the relationship between recurrent trauma and hallucinations. **p < .01; ***p < .001; ^ns^ nonsignificant.

As shown in Figure 2, in the path recurrent trauma‐ > disorganized attachment‐ > hallucinations, recurrent trauma had a significant positive effect on disorganized attachment (a^1^: *b* = 0.639, *p* = .001), while disorganized attachment did not have a significant effect on hallucinations (b^2^: *b* = 0.008, *p* = .647).

In the path recurrent trauma‐ > dissociation‐ > hallucinations, recurrent trauma did not significantly affect dissociation (a^2^: *b* = 0.119, *p* = .169); however; dissociation had a significant positive effect on hallucinations (b^2^: *b* = 0.140, *p* < .001).

In the path recurrent trauma‐ > disorganized attachment‐ > dissociation‐ > hallucinations, recurrent trauma positively impacted disorganized attachment (a^1^: *b* = 0.639, *p* = .001), disorganized attachment positively impacted dissociation (a^3^: *b* = 0.189, *p* < .001), and dissociation positively impacted hallucinations (b^2^: *b* = 0.140, *p* < .001). Furthermore, the direct effect of recurrent trauma on hallucinations was not significant (c: *b* = −0.012, *p* = .644). This indicates that the sequence of disorganized attachment and dissociation fully mediated the relationship between recurrent trauma and hallucinations.

#### Delusions

**FIGURE 3:**
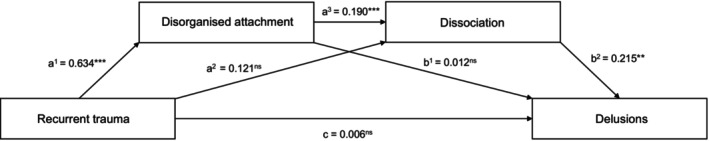
Mediation model testing if disorganized attachment and dissociation serially mediate the relationship between recurrent trauma and delusions, while controlling for gender. **p < .01; ***p < .001; ^ns^ nonsignificant.

As shown in Figure 3, in the path recurrent trauma‐ > disorganized attachment‐ > delusions, recurrent trauma had a significant positive effect on disorganized attachment while controlling for gender (a^1^: *b* = 0.634, *p* = .001), while disorganized attachment did not have a significant effect on delusions (b^1^: *b* = 0.012, *p* = .690).

In the path recurrent trauma‐ > dissociation‐ > delusions, recurrent trauma did not significantly affect dissociation while controlling for gender (a^2^: *b* = 0.121, *p* = .168); however, dissociation had a significant positive effect on delusions (b^2^: *b* = 0.215, *p* = .001).

In the path recurrent trauma‐ > disorganized attachment‐ > dissociation‐ > delusions, recurrent trauma positively impacted disorganized attachment while controlling for gender (a^1^: *b* = 0.634, *p* = .001), disorganized attachment positively impacted dissociation (a^3^: *b* = 0.190, *p* < .001), and dissociation positively impacted delusions (b^2^: *b* = 0.215, *p* = .001). Further, the direct effect of recurrent trauma on delusions was not significant (c: *b* = 0.006, *p* = .903). This indicates that the sequence of disorganized attachment and dissociation fully mediated the relationship between recurrent trauma and delusions.

## DISCUSSION

The primary aim of this study was to examine the overall predictive value of a model of trauma and social pathways to psychosis in people with a psychosis‐related diagnosis. Findings indicated that recurrent trauma, insecure attachment and dissociation significantly predicted positive symptoms of psychosis, while social rank did not. A secondary aim was to delineate specific pathways between trauma and psychosis. Serial mediation analysis indicated that the sequence of disorganized attachment and dissociation fully mediated the relationship between recurrent trauma and positive symptoms of psychosis. As social rank was not significantly associated with trauma or psychosis, it was not examined in the mediation analyses.

### Overall predictive value of the model

Hierarchical regressions indicated that recurrent trauma, insecure attachment and dissociation predicted a large amount (23.2%) of the variance in positive symptoms. While the individual role of these processes is relatively well evidenced (Bloomfield et al., 2021; Mason et al., 2023; Williams et al., 2018), this is one of the few studies that has demonstrated the overall predictive value of this cluster of trauma‐related variables (Mertens et al., 2021; Pearce et al., 2017; Strachan et al., 2023). Further, prior studies that have examined these variables have relied on non‐clinical or online samples. Extending these findings to people who are actively engaged in mental health services for psychosis‐related diagnoses is an important step forward, as it demonstrates the relevance of these processes to the cohort of people who are most likely to be impacted by the clinical implications that follow from them.

### Social rank was not a significant predictor of psychosis

Despite correlating with both attachment and dissociation, social rank was not a significant predictor of psychosis. While it may be that social rank is less influential than the other processes examined, there are methodological and conceptual factors to consider. Firstly, the SCS may not have adequately captured social rank, as it does not assess the associated behavioural or emotional patterns (submissive behaviour, shame). Conceptually, the measurement of shame may be particularly relevant, given that the current theory posits that shame may predict dissociative responses and, ultimately, psychosis. Although under‐researched, the relevance of shame in these interactions has been illustrated in several prior studies. Wood and Irons (2016) examined social rank and external shame in an FEP sample, and found that positive symptoms were associated with external shame, but not social rank (as measured by the SCS). More recently, Heriot‐Maitland et al. (2024) examined interactions between shame, dissociation and psychotic‐like experiences in a non‐clinical sample. The study found that, at high levels of external shame, high levels of dissociation led to higher levels of psychotic‐like experiences. Thus, including a measure of shame, or alternative measures of social rank, may have shed greater light on potential social rank‐psychosis interactions in the current study.

### Associations between trauma and psychosis

Adverse life experiences were frequently reported in our sample, with participants reporting a mean exposure to eight adverse events, a figure comparable to prior studies that have used the TALE in a clinical setting (Campodonico et al., 2021; Carr et al., 2018b). Despite these rates, total trauma exposure was not associated with psychosis. Instead, only trauma that was recurrent in nature (arguably, an indicator of more severe trauma) correlated with positive symptoms. This is consistent with recent findings that trauma severity, not exposure, predicts psychosis (Mason et al., 2023), as well as evidence that psychosis is more strongly associated with cPTSD than PTSD (Mason et al., 2023; Panayi et al., 2022). This highlights the importance of considering the nature and severity of trauma, rather than exposure alone, in pathways to psychosis. Additionally, the strength of associations between lifetime rates of recurrent trauma and other variables of interest extends on prior research that has focused exclusively on childhood trauma (e.g., Varese, Barkus, & Bentall, 2012; Varese, Smeets, et al., 2012), suggesting that the dose–responsive relationship evidenced between childhood trauma and psychosis may be applicable across the lifespan.

### Attachment and dissociation as serial mediators

Serial mediation analysis indicated that recurrent trauma did not independently predict psychosis. Instead, it exerted its influence on psychosis through the sequence of disorganized attachment and dissociation. Further, disorganized attachment and dissociation fully mediated the relationship between recurrent trauma and both hallucinations and delusions when each symptom domain was entered as the dependent variable. These results build on those from our primary analysis; while the regression model indicated that recurrent trauma was a significant predictor of psychosis, serial mediation illustrated that trauma itself does not lead to psychosis, rather, it predicts a sequence of processes that do.

Although disorganized attachment was examined in the serial mediation analyses, it was not a significant independent predictor of psychosis in our primary regression model. Instead of the attachment subscales, only avoidant attachment was a significant predictor. There are several factors to consider when interpreting this. Firstly, the PAM‐R is a continuous measure of attachment and there were significant correlations between subscales, meaning that many participants are likely to have had high scores on multiple subscales. Secondly, insecure attachment accounted for small to medium amounts of variance in psychosis overall. Consequently, attachment subscales are likely to move in and out of significance based on sample size and statistics used. The clinical significance of one insecure attachment pattern over another, therefore, should not be overemphasized based on these results.

The current findings are consistent with prior studies that have demonstrated the role of insecure attachment and dissociation in pathways to unusual perceptual experiences and paranoia (e.g., Mertens et al., 2021; Pearce et al., 2017). Furthermore, although we did not examine individual SAPS items, the breadth of symptoms included in our models suggests that the influence of attachment and dissociation may extend to a wider range of psychotic experiences (e.g., non‐paranoid delusions, visual hallucinations). While these findings support prominent models of trauma and psychosis (Berry et al., 2017; Hardy, 2017; Heriot‐Maitland et al., 2022), it is important to note that a range of variables that have been causally implicated were not examined. For example, the CAV model (Berry et al., 2017) emphasizes the role of negative schemas in pathways between disorganized attachment and voice‐hearing, and this evidence base has recently been extended to paranoia (Humphrey et al., 2022). Similarly, Hardy's model (2017) highlights the role of trauma‐related beliefs and affective dysregulation, both of which have relevance to attachment and dissociation, and evidence suggests may be the most influential links between symptoms of PTSD and psychosis (Hardy et al., 2021).

### Limitations

While the study was powered to detect small effect sizes using regression analysis, it only had precision to detect medium to large effect sizes in the mediation analyses. However, mediation analyses were based on *a‐priori* hypotheses that are in line with theoretical accounts of psychosis, as well as evidence from prior non‐clinical samples. The small sample size also meant that potentially relevant socio‐demographic or clinical covariates were not controlled for. In the serial mediation analysis, delusions and hallucinations were not entered as covariates in each respective analysis, which may have confounded findings. This decision was made, however, given that each symptom may form part of the causal pathway to another and controlling for them may have removed relevant variance in outcomes. The cross‐sectional nature of the study also means that causality between variables in the model cannot be determined. Furthermore, the method of recruitment may have introduced selection biases, as people with attachment insecurity are less likely to engage with mental health services (McGonagle et al., 2021). Lastly, the sample was predominantly white‐Irish, which limits the generalizability of findings as evidence indicates that psychosis is higher among individuals from minority ethnic backgrounds (Qassem et al., 2015).

### Clinical implications

Findings highlight the importance of dissociation and attachment in our formulation and treatment of psychosis. While current guidelines recommend CBT for people with psychosis (NICE, 2013), manualized CBTp often does not sufficiently address these processes (Hardy et al., 2024; Newman‐Taylor & Bentall, 2024). A promising stream of research is emerging that demonstrates how CBTp interventions can be modified to attend to these processes, however (e.g., Newman‐Taylor & Bentall, 2024; Varese et al., 2021). For example, mindfulness techniques can increase awareness of, and control over, dissociation (Newman‐Taylor & Sambrook, 2013), while attachment‐focused imagery can be incorporated as a way to increase felt security (Airey et al., 2023; Newman‐Taylor, 2020). There is also extensive trauma literature that can be used to support people experiencing dissociation (e.g., Chessell et al., 2019; Schauer & Elbert, 2010). Lastly, there is mounting evidence for the use of compassion‐focused therapy in psychosis (Heriot‐Maitland, 2024), which targets both attachment and dissociation through its focus on developing care‐focused systems and experiences of social safeness.

Attachment security can also be fostered at a service level. Attachment difficulties in people with psychosis are associated with poorer engagement with mental health services (McGonagle et al., 2021), and it is common for negative attachment patterns experienced by clients to get played out on a larger scale in interactions with teams (Priebe et al., 2011). It may be useful for services to consider ways in which they can increase their awareness of how attachment patterns influence engagement, and promote safeness through warmth, sensitivity, and consistency in care.

### Future research

Replication studies with larger clinical samples are needed, as well as longitudinal studies to confirm the direction of pathways identified in this study. Lager samples would also allow for the use of more sophisticated modelling techniques. Pathways to psychosis are complex and, for example, there is evidence for a range of additional cognitive and affective mechanisms in pathways between attachment and paranoia alone (e.g., Sood et al., 2022). Techniques such as latent class analysis or network analysis may have the potential to better differentiate routes. To further elucidate social pathways to psychosis, future studies should include a measure of shame and perhaps consider alternative ways to assess social rank.

## CONCLUSIONS

The current study examined a model of trauma and social pathways to psychosis in people with a psychosis‐related diagnosis. Overall, results support the role of recurrent trauma, attachment and dissociation in the development of positive symptoms. Results did not support the social rank component of this model, however. These findings extend on prior research by providing evidence for trauma pathways to psychosis in a clinical sample, and highlight several important considerations for the future exploration of social pathways to psychosis.

## AUTHOR CONTRIBUTIONS

**Shelley Grady:** Conceptualization; investigation; writing – original draft; methodology; formal analysis; project administration. **Niall Crowley:** Resources; methodology; project administration. **Seamus Scott:** Investigation; project administration; methodology. **Charles Ifegwu Ndukwe:** Investigation. **Rebecca Donohoe:** Project administration. **Keith Gaynor:** Conceptualization; methodology; writing – review and editing; supervision.

## FUNDING INFORMATION

There was no funding received for this research. The first author completed this research as part of her Doctorate in Clinical Psychology at University College Dublin and is sponsored by the Health Service Executive while completing this training.

## CONFLICT OF INTEREST STATEMENT

No conflicts of interest were identified.

## ETHICS STATEMENT

Ethical approval for this study was obtained from University College Dublin and HSE Research Ethics Committees (REC no: RRECB0722SG1). Participation was remunerated through a prize draw (€100). All procedures were carried out in accordance with institutional and national ethical guidelines for human studies, and the guidelines proposed in the Declaration of Helsinki.

## Supporting information


Tables S1–S13.

## Data Availability

The data that support the findings of this study are available from the corresponding author upon reasonable request.
