# Supplementary material for: Trauma and social pathways to psychosis: Examining the role of attachment, social rank and dissociation in a clinical sample
Source: Br J Clin Psychol. 2024 Oct 29;64(2):385–402. doi: 10.1111/bjc.12511 (PMC12057311; doi:10.1111/bjc.12511)
Supplement: Supplementary file 1 — Tables S1–S13. [file BJC-64-385-s001.docx]

| Supplementary Table 1.  *Rates of trauma and abuse type (N = 71)* | | | |
| --- | --- | --- | --- |
|  |  | **Frequency n (%)** | **Repeated exposure n (%)** |
| **Individual TALE Items** |  |  |  |
| 1. Exposure to war (n=69) | Lifetime | 3 (4.4) | 2 (2.9) |
|  | Child | 3 (4.4) | 2 (2.9) |
|  | Adult | 1(1.4) | 1 (1.4) |
| 1. Permanent separation/loss (n=69) | Lifetime | 36 (52.2) | 23 (33.3) |
|  | Child | 15 (21.7) | 12 (17.4) |
|  | Adult | 26 (37.7) | 16 (23.2) |
| 1. Period of separation caregiver (n=71) | Lifetime | 24 (33.8) | 6 (8.5) |
|  | Child | 9 (12.7) | 2 (2.8) |
|  | Adult | 1. 22.5) | 5 (7.0) |
| 1. Sudden move/change (n=70) | Lifetime | 34 (48.6) | 18 (25.7) |
|  | Child | 14 (20.0) | 11 (15.7) |
|  | Adult | 1. 31.4) | 9 (12.9) |
| 1. Bullying/harassment (n=71) | Lifetime | 41 (57.7) | 34 (47.9) |
|  | Child | 35 (49.3) | 32 (45.1) |
|  | Adult | 1. 22.5) | 12 (16.9) |
| 1. Discrimination (n=69) | Lifetime | 30 (43.5) | 28 (40.6) |
|  | Child | 19 (27.5) | 19 (27.5) |
|  | Adult | 1. 29.0) | 18 (26.1) |
| 1. Emotional abuse (someone close) (n=68) | Lifetime | 42 (61.8) | 34 (50.0) |
|  | Child | 29 (42.6) | 26 (38.2) |
|  | Adult | 28 (41.2) | 23 (33.8) |
| 1. Physical abuse (someone close) (n=70) | Lifetime | 30 (42.9) | 24 (34.3) |
|  | Child | 20 (28.6) | 17 (24.3) |
|  | Adult | 14 (20.0) | 9 (15.7) |
| 1. Witnessing violence at home (70) | Lifetime | 43 (61.4) | 38 (54.3) |
|  | Child | 37 (52.9) | 34 (48.6) |
|  | Adult | 12 (17.1) | 10 (14.3) |
| 1. Violence outside the home (n=69) | Lifetime | 25 (36.2) | 19 (27.6) |
|  | Child | 12 (17.4) | 11 (15.9) |
|  | Adult | 16 (23.2) | 11 (15.9) |
| 1. Emotional neglect childhood (n=71) |  | 29 (40.8) | 28 (39.4) |
| 1. Physical neglect childhood (n=71) |  | 13 (18.3) | 17 (16.9) |
| 1. Unwanted sexual contact <16 (n=71) |  | 21 (29.6) | 9 (15.5) |
| 1. Unwanted sexual contact >16 (n=71) |  | 19 (26.8) | 9 (11.3) |
| 1. Psychosis (symptoms) (n=71) | Lifetime | 46 (64.8) | 42 (59.2) |
|  | Child | 13 (18.3) | 13 (18.3) |
|  | Adult | 40 (56.3) | 36 (50.7) |
| 1. Psychosis (unusual behaviours) (n=70) | Lifetime | 29 (41.4) | 22 (31.4) |
|  | Child | 5 (7.1) | 4 (5.7) |
|  | Adult | 27 (38.6) | 21 (30.0) |
| 1. Psychosis (treatment) ( (n=70) | Lifetime | 37 (52.9) | 27 (38.6) |
|  | Child | 4 (5.7) | 4 (5.7) |
|  | Adult | 35 (50.0) | 14 (35.7) |
| 1. Experiences with health/justice (n=70) | Lifetime | 21 (30.0) | 9 (12.9) |
|  | Child | 4 (5.7) | 2 (2.9) |
|  | Adult | 19 (27.1) | 9 (12.9) |
| 1. Accidents and illnesses (n=70) | Lifetime | 12 (17.1) | 4 (5.7) |
|  | Child | 2 (2.9) | 2 (2.9) |
|  | Adult | 11 (15.7) | 3 (4.3) |
| Note: Lifetime scores are based on overall exposure (childhood, adulthood or both). | | | |

| Supplementary Table 2.  *Independent samples t-test examining differences between primary measures based on identified gender* | | | | | |
| --- | --- | --- | --- | --- | --- |
|  | Males  (n = 42) | Females  (n = 29) |  |  |  |
| Measure | *M (SD)* | *M (SD)* | df | *t* | *p* |
| TALE total | 7.99 (4.11) | 8.38 (3.55) | 69 | .419 | .677 |
| TALE recurrent | 6.21 (3.89) | 5.36 (3.16) | 67.09 | -1.016 | .313 |
| SAPS total | 4.67 (3.35) | 3.21 (3.24) | 69 | -1.829 | .072 |
| SAPS hallucinations^†^ | 0.67 (0.78) | 0.76 (0.83) | 69 | 0.458 | .649 |
| SAPS delusions | 1.74 (1.449) | 0.97 (1.295) | 69 | -2.304 | **.024** |
| DES-II^†^ | 6.37 (2.57) | 6.23 (2.96) | 69 | -0.219 | .828 |
| PAM anxious | 8.36 (4.30) | 10.34 (5.04) | 69 | 1.778 | .080 |
| PAM avoidant | 9.40 (3.04) | 8.55 (3.76) | 69 | -1.047 | .299 |
| PAM disorganised | 8.94 (6.00) | 8.14 (6.13) | 69 | -0.550 | .584 |
| SCS | 53.89 (19.89) | 53.27 (20.24) | 69 | -0.128 | .898 |
| Significant scores in bold.  ^†^Raw score is log transformed. | | | | | |

| Supplementary Table 3.  *Independent samples t-test examining differences between primary measures based on ethnic group* | | | | | |
| --- | --- | --- | --- | --- | --- |
|  | White-Irish  (n = 62) | Minority/  Non-Irish  (n = 9) |  |  |  |
| Measure | *M (SD)* | *M (SD)* | df | *t* | *p* |
| TALE total | 7.90 (3.94) | 9.88 (2.97) | 69 | -1.450 | .152 |
| TALE recurrent | 5.51 (3.59) | 8.26 (2.83) | 69 | -2.196 | **.031** |
| SAPS total | 3.645 (3.240) | 7.00 (2.784) | 69 | -2.948 | **.004** |
| SAPS hallucinations^†^ | 0.614 (0.786) | 1.35 (0.58) | 12.74 | -3.377 | **.005** |
| SAPS delusions | 1.34 (1.43) | 2.00 (1.41) | 69 | -1.302 | .197 |
| DES-II^†^ | 5.99 (2.64) | 8.54 (2.28) | 69 | -2.753 | **.008** |
| PAM anxious | 9.16 (4.73) | 9.24 (4.60) | 69 | -0.046 | .963 |
| PAM avoidant | 8.88 (3.35) | 10.26 (3.30) | 69 | -1.159 | .250 |
| PAM disorganised | 8.26 (6.06) | 11.07 (5.48) | 69 | -1.315 | .193 |
| SCS | 53.03 (20.04) | 57.78 (19.46) | 69 | -0.666 | .508 |
| Significant scores in bold.  ^†^Raw score is log transformed. | | | | | |

| Supplementary Table 4.  *Independent samples t-test examining differences between primary measures based on relationship status* | | | | | |
| --- | --- | --- | --- | --- | --- |
|  | Single  (n = 55) | In a relationship  (n = 16) |  |  |  |
| Measure | *M (SD)* | *M (SD)* | df | *t* | *p* |
| TALE total | 7.86 (3.79) | 9.23 (4.13) | 69 | 1.148 | .255 |
| TALE recurrent | 5.93 (3.66) | 5.63 (3.50) | 69 | -0.295 | .769 |
| SAPS total | 4.31 (3.33) | 3.35 (3.45) | 69 | -1.111 | .270 |
| SAPS hallucinations^†^ | 0.71 (0.81) | 0.71 (0.78) | 69 | 0.00 | 1.00 |
| SAPS delusions | 1.44 (1.41) | 1.38 (1.45) | 69 | -0.150 | .881 |
| DES-II^†^ | 6.31 (2.54) | 6.31 (3.35) | 20.29 | -0.006 | .996 |
| PAM anxious | 8.91 (4.54) | 10.06 (5.20) | 69 | 0.861 | .392 |
| PAM avoidant | 9.40 (3.24) | 7.88 (3.56) | 69 | -1.616 | .111 |
| PAM disorganised | 9.16 (6.08) | 6.75 (5.63) | 69 | -1.416 | .161 |
| SCS | 54.65 (20.17) | 50.13 (19.10) | 69 | -.800 | .427 |
| Significant scores in bold.  ^†^Raw score is log transformed. | | | | | |

| Supplementary Table 5.  *Independent samples t-test examining differences between primary measures based on medication status* | | | | | |
| --- | --- | --- | --- | --- | --- |
|  | Anti-psychotic medication  (n = 67) | No anti-psychotic medication  (n = 4) |  |  |  |
| Measure | *M (SD)* | *M (SD)* | df | *t* | *p* |
| TALE total | 7.99 (3.93) | 11.25 (0.50) | 41.04 | 5.979 | **<.001** |
| TALE recurrent | 9.5 (1.91) | 5.64 (3.60) | 68 | 2.116 | **.038** |
| SAPS total | 4.08 (3.45) | 4.5 (1.91) | 68 | 0.242 | .809 |
| SAPS hallucinations^†^ | 0.68 (0.81) | 1.06 (0.71) | 68 | 0.918 | .362 |
| SAPS delusions | 1.44 (1.47) | 1.25 (0.96) | 68 | -0.254 | .801 |
| DES-II^†^ | 6.25 (2.68) | 8.18 (2.74) | 68 | 1.393 | .168 |
| PAM anxious | 9.17 (4.62) | 11.00 (5.35) | 68 | 0.764 | .448 |
| PAM avoidant | 9.24 (3.24) | 8.00 (3.37) | 68 | -0.742 | .461 |
| PAM disorganised | 8.62 (5.90) | 10.00 (8.91) | 68 | 0.444 | .659 |
| SCS | 52.89 (19.91) | 59.50 (19.21) | 68 | 0.645 | .521 |
| Significant scores in bold.  ^†^Raw score is log transformed. | | | | | |

| Supplementary Table 6.  *Independent samples t-test examining differences between primary measures based on employment status* | | | | | |
| --- | --- | --- | --- | --- | --- |
|  | **Working/**  **Studying**  **(n = 30)** | **Unemployed (n = 41)** |  |  |  |
| Measure | *M (SD)* | *M (SD)* | df | *t* | *p* |
| TALE total | 8.09 (3..89) | 8.19 (3.91) | 69 | -0.114 | .910 |
| TALE recurrent | 5.40 (3.30) | 6.20 (3.82) | 69 | -0.923 | .359 |
| SAPS total | 3.47 (3.20) | 4.51 (3.44) | 69 | -1.301 | .198 |
| SAPS hallucinations^†^ | 0.55 (0.71) | 0.82 (0.84) | 67.50 | -1.422 | .160 |
| SAPS delusions | 1.30 (1.37) | 1.51 (1.49) | 69 | -0.614 | .541 |
| DES-II^†^ | 5.92 (2.20) | 6.60 (3.03) | 69 | -1.085 | .282 |
| PAM anxious | 8.91 (4.06) | 9.37 (5.13) | 69 | -0.410 | .683 |
| PAM avoidant | 8.58 (2.84) | 9.40 (3.67) | 69 | -1.017 | .313 |
| PAM disorganised | 7.80 (5.57) | 9.21 (6.34) | 69 | -0.970 | .335 |
| SCS | 54.39 (19.21) | 53.09 (20.60) | 69 | 0.271 | .787 |
| Significant scores in bold.  ^†^Raw score is log transformed. | | | | | |

| Supplementary Table 7.  *Independent samples t-test examining differences between primary measures based on current mental health service* | | | | | |
| --- | --- | --- | --- | --- | --- |
|  | **Secondary care**  **(n = 61)** | **Tertiary care**  **(n = 10)** |  |  |  |
| Measure | *M (SD)* | *M (SD)* | df | *t* | *p* |
| TALE total | 8.31 (3.78) | 7.16 (4.52) | 69 | 0.872 | .386 |
| TALE recurrent | 5.90 (3.40) | 5.64 (4.91) | 69 | -0.207 | .837 |
| SAPS total | 4.02 (3.26) | 4.40 (4.09) | 69 | -0.332 | .741 |
| SAPS hallucinations^†^ | 0.65 (0.77) | 1.03 (0.91) | 69 | 1.389 | .169 |
| SAPS delusions | 1.44 (1.42) | 1.30 (1.57) | 69 | 0.290 | .773 |
| DES-II^†^ | 6.31 (2.74) | 6.33 (2.72) | 69 | 0.024 | .981 |
| PAM anxious | 9.42 (4.83) | 7.70 (3.53) | 69 | 1.076 | .286 |
| PAM avoidant | 8.76 (3.26) | 10.84 (3.53) | 69 | -1.851 | .068 |
| PAM disorganised | 8.36 (5.88) | 10.14 (7.00) | 69 | -0.863 | .391 |
| SCS | 53.22 (19.80) | 56.14 (21.36) | 69 | -0.427 | .671 |
| Significant scores in bold.  ^†^Raw score is log transformed. | | | | | |

| Supplementary Table 8.  *One-way analysis of variance using level of education as the criterion* | | | | | | | | |
| --- | --- | --- | --- | --- | --- | --- | --- | --- |
|  | ≤ Junior cert/  equivalent  (n = 16) | Leaving cert/  equivalent  (n = 14) | College  (n = 26) | Undergrad  (n = 8) | ≥ Postgrad  (n = 7) |  |  |  |
| Measure | *M (SD)* |  |  |  |  | *F*  (4, 66) | *p* | *n^2^* |
| TALE total | 7.82 (3.45) | 7.75 (4.29) | 8.31 (4.43) | 7.88 (3.64) | 9.43 (2.30) | 0.267 | .898 | .016 |
| TALE recurrent | 5.56 (3.33) | 6.58 (4.34) | 5.62 (3.69) | 5.13 (2.47) | 6.86 (3.98) | 0.401 | .808 | .024 |
| SAPS total | 4.94 (3.96) | 4.93 (3.10) | 3.58 (3.06) | 3.38 (3.42) | 3.00 (3.51) | 0.982 | .474 | .051 |
| SAPS hallucinations^†^ | 0.66 (0.90) | 0.81 (0.87) | 0.70 (0.75) | 0.66 (0.71) | 0.68 (0.89) | 0.080 | .988 | .005 |
| SAPS delusions | 1.75 (1.61) | 1.71 (1.27) | 1.23 (1.42) | 1.25 (1.39) | 1.00 (1.53) | 0.640 | .636 | .037 |
| DES-II^†^ | 5.71 (3.10) | 6.77 (2.37) | 6.49 (2.84) | 6.17 (2.33) | 6.28 (2.72) | 0.319 | .864 | .019 |
| PAM anxious | 8.52 (3.77) | 9.14 (4.40) | 9.77 (5.64) | 7.63 (5.64) | 10.29 (4.64) | 0.486 | .746 | .029 |
| PAM avoidant | 10.97 (3.12) | 9.79 (3.11) | 7.70 (3.40) | 8.75 (2.25) | 8.57 (3.87) | 2.852 | **.030^§^** | .147 |
| PAM disorganised | 9.57 (5.52) | 10.89 (5.92) | 7.88 (5.61) | 6.00 (5.73) | 7.57 (8.64) | 1.134 | .348 | .064 |
| SCS | 53.21 (26.16) | 54.55 (17.65) | 55.04 (19.42) | 57.00 (13.34) | 43.71 (17.64) | 0.519 | .722 | .031 |
| ^†^Raw score is log transformed.  Significant scores in bold.  ^§^Ducan post-hoc tests indicated no specific differences between groups. | | | | | | | | |

| Supplementary Table 9.  *One-way analysis of variance using psychosis-related diagnosis as the criterion* | | | | | | | | | | |
| --- | --- | --- | --- | --- | --- | --- | --- | --- | --- | --- |
|  | Schizophrenia  (n = 28) | Schizoaffective  (n = 14) | Delusional disorder (n = 2) | FEP  (n = 15) | PDNOS  (n = 3) | Bipolar with psychosis  (n = 5) | Dep with psychosis  (n = 4) |  |  |  |
| Measure | *M (SD)* |  |  |  |  |  |  | *F*  (6, 64) | *p* | *n^2^* |
| TALE total | 8.11 (3.90) | 8.07 (4.45) | 6.00 (0.00) | 8.60 (4.39) | 8.00 (2.65) | 8.40 (4.16) | 7.86 (2.39) | 0.135 | .991 | .012 |
| TALE recurrent | 5.81 (3.51) | 5.86 (4.42) | 3.50 (3.54) | 6.76 (3.82) | 7.00 (1.73) | 4.40 (2.97) | 5.04 (2.93) | 0.498 | 0.808 | .045 |
| SAPS total | 5.18 (3.79) | 2.21 (2.69) | 6.50 (0.71) | 4.33 (2.89) | 5.33 (3.06) | 2.20 (2.28) | 2.00 (2.31) | 2.194 | .055 | .171 |
| SAPS hallucinations^†^ | 1.00 (0.86) | 0.20 (0.51) | 1.21 (0.29) | 0.69 (0.80) | 0.80 (0.73) | 0.57 (0.77) | 0.35 (0.71) | 2.019 | .076 | .159 |
| SAPS delusions | 1.71 (1.54) | 0.71 (1.20) | 4.00 (0.00) | 1.47 (1.19) | 2.33 (1.53) | 0.40 (0.55) | 1.00 (1.16) | 2.962 | **.013^§^** | .217 |
| DES-II^†^ | 6.67 (2.80) | 5.15 (3.02) | 9.96 (1.35) | 6.75 (1.96) | 6.15 (4.50) | 6.56 (2.19) | 4.32 (1.05) | 1.616 | .157 | .132 |
| PAM anxious | 8.25 (4.13) | 10.86 (4.24) | 13.00 (0.00) | 8.68 (5.12) | 9.00 (7.55) | 8.60 (4.98) | 10.50 (7.19) | 0.783 | .586 | .068 |
| PAM avoidant | 10.31 (2.83) | 7.07 (3.47) | 7.00 (2.83) | 9.14 (2.75) | 9.33 (7.23) | 8.80 (2.59) | 8.00 (4.34) | 1.787 | .116 | .144 |
| PAM disorganised | 8.82 (6.06) | 8.21 (6.46) | 7.00 (5.66) | 7.64 (5.29) | 12.67 (10.02) | 9.00 (5.61) | 9.50 (7.72) | 0.329 | .919 | .030 |
| SCS | 53.96 (20.60) | 49.43 (15.40) | 43.50 (4.95) | 55.40 (22.48) | 71.00 (24.98) | 51.40 (23.89) | 54.25 (19.10) | 0.582 | .743 | .052 |
| ^†^Raw score is log transformed.  Significant scores in bold.  ^§^Ducan post-hoc tests indicated no specific differences between groups. | | | | | | | | | | |

| Supplementary Table 10.  *Pearson’s correlations between primary measures and age* | | | |
| --- | --- | --- | --- |
| Measure | *df* | *r* | *p* |
| TALE total | 69 | 0.037 | .758 |
| TALE recurrent | 69 | 0.034 | .777 |
| SAPS total | 69 | -0.147 | .698 |
| SAPS hallucinations^†^ | 69 | -0.149 | .214 |
| SAPS delusions | 69 | -0.068 | .572 |
| DES-II^†^ | 69 | -0.247 | **.037** |
| PAM anxious | 69 | -0.115 | .339 |
| PAM avoidant | 69 | -0.066 | .583 |
| PAM disorganised | 69 | 0.004 | .977 |
| SCS | 69 | 0.076 | .531 |
| ^†^Raw score is log transformed.  Significant scores in bold. | | | |

| Supplementary Table 11.  *Pearson’s correlations between primary measures and duration of contact with mental health services for a psychosis-related diagnosis* | | | |
| --- | --- | --- | --- |
| Measure | *df* | *r* | *p* |
| TALE total | 69 | 0.154 | .201 |
| TALE recurrent | 69 | 0.305 | **.010** |
| SAPS total | 69 | 0.065 | .592 |
| SAPS hallucinations^†^ | 69 | -0.232 | .052 |
| SAPS delusions | 69 | 0.025 | .838 |
| DES-II^†^ | 69 | -0.094 | .434 |
| PAM anxious | 69 | -0.029 | .809 |
| PAM avoidant | 69 | 0.106 | .381 |
| PAM disorganised | 69 | -0.003 | .978 |
| SCS | 69 | 0.040 | .744 |
| ^†^Raw score is log transformed.  Significant scores in bold. | | | |

| Supplementary table 12.  *Hierarchical linear regression predicting SAPS hallucinations* | | | | | | | |
| --- | --- | --- | --- | --- | --- | --- | --- |
| Predictors | Adjusted R^2^ | ΔR^2^ | *B* | *SE B* | *b* | Model F *(df*) | *p* |
| **Step 1** | .000 | .014 |  |  |  | .998 (1, 69) | .321 |
| Recurrent trauma |  |  | .026 | .026 | .119 |  |  |
| **Step 2** | .074 | .113 |  |  |  | 2.840 (3, 66) | .045* |
| Recurrent trauma |  |  | .010 | .028 | .045 |  |  |
| PAM anxious |  |  | -.012 | .026 | -.070 |  |  |
| PAM avoidant |  |  | .057 | .029 | .240* |  |  |
| PAM disorganised |  |  | .029 | .022 | .219 |  |  |
| **Step 3** | .062 | .002 |  |  |  | .123 (1, 65) | .727 |
| Recurrent trauma |  |  | .010 | .028 | .045 |  |  |
| PAM anxious |  |  | -.014 | .027 | -.082 |  |  |
| PAM avoidant |  |  | .057 | .029 | .238 |  |  |
| PAM disorganised |  |  | .027 | .023 | .205 |  |  |
| Social rank |  |  | -.002 | .005 | -.047 |  |  |
| **Step 4** | .224 | .161 |  |  |  | 14.558 (1, 64) | <.001*** |
| Recurrent trauma |  |  | -.007 | .026 | -.031 |  |  |
| PAM anxious |  |  | -.028 | .025 | -.164 |  |  |
| PAM avoidant |  |  | .045 | .027 | .188 |  |  |
| PAM disorganised |  |  | .012 | .021 | .087 |  |  |
| Social rank |  |  | -.001 | .005 | -.022 |  |  |
| Dissociation |  |  | .139 | .037 | .474*** |  |  |
| **p* < .05; ***p* < .01; ****p* < .001 | | |  |  |  |  |  |

| Supplementary table 13.  *Hierarchical linear regression predicting SAPS delusions while controlling for gender* | | | | | | | |
| --- | --- | --- | --- | --- | --- | --- | --- |
| Predictors | Adjusted R^2^ | ΔR^2^ | *B* | *SE B* | *b* | Model F *(df*) | *p* |
| **Step 1** | .058 | .071 |  |  |  | 5.309 (1, 69) | .024* |
| Gender |  |  | .773 | .335 | .267* |  |  |
| **Step 2** | .063 | .018 |  |  |  | 1.349 (1, 68) | .250 |
| Gender |  |  | .727 | .337 | .251* |  |  |
| Recurrent trauma |  |  | .054 | .046 | .135 |  |  |
| **Step 3** | .084 | .060 |  |  |  | 1.516 (3, 65) | .219 |
| Gender |  |  | .745 | .356 | .258* |  |  |
| Recurrent trauma |  |  | .023 | .050 | .058 |  |  |
| PAM anxious |  |  | .029 | .049 | .096 |  |  |
| PAM avoidant |  |  | .052 | .052 | .122 |  |  |
| PAM disorganised |  |  | .028 | .040 | .118 |  |  |
| **Step 4** | 0.82 | .012 |  |  |  | .885 (1, 64) | .350 |
| Gender |  |  | .740 | .356 | .256* |  |  |
| Recurrent trauma |  |  | .023 | .050 | .058 |  |  |
| PAM anxious |  |  | .020 | .050 | .065 |  |  |
| PAM avoidant |  |  | .051 | .052 | .119 |  |  |
| PAM disorganised |  |  | .019 | .041 | .081 |  |  |
| Social rank |  |  | -.009 | .010 | -.124 |  |  |
| **Step 5** | .189 | .110 |  |  |  | 9.454 (1, 63) | .003** |
| Gender |  |  | .720 | .335 | .249 |  |  |
| Recurrent trauma |  |  | -.002 | .048 | -.004 |  |  |
| PAM anxious |  |  | -.002 | .048 | -.005 |  |  |
| PAM avoidant |  |  | .033 | .049 | .078 |  |  |
| PAM disorganised |  |  | -.003 | .039 | -.014 |  |  |
| Social rank |  |  | -.008 | .009 | -.104 |  |  |
| Dissociation |  |  | .206 | .067 | .391** |  |  |
| **p* < .05; ***p* < .01; ****p* < .001 | | |  |  |  |  |  |
